# Molecular Characterization of *Echinococcus vogeli* from Human Case, Colombia, 2024

**DOI:** 10.3201/eid3108.250361

**Published:** 2025-08

**Authors:** Jorge Alfredo Morcillo Muñoz, Diego Andrés Rodríguez-Lugo, Javier Hernández Moreno, María Helena Astorquiza, Luz Helena Patiño, Tatiana Marcela Cáceres, Angie Lorena Ramírez, Juan David Ramírez, Álvaro A. Faccini-Martínez

**Affiliations:** Universidad Nacional de Colombia, Bogotá, Colombia (J.A. Morcillo Muñoz, D.A. Rodríguez-Lugo); Hospital Militar Central, Bogotá (J. Hernández Moreno, M.H. Astorquiza, Á.A. Faccini-Martínez); Universidad del Rosario, Bogotá (L.H. Patiño, T.M. Cáceres, A.L. Ramírez, J.D. Ramírez); University of South Florida, Tampa, Florida, USA (J.D. Ramírez); Universidad Militar Nueva Granada, Bogotá (Á.A. Faccini-Martínez)

**Keywords:** echinococcosis, *Echinococcus vogeli*, parasites, zoonoses, tapeworm, hepatic, Colombia

## Abstract

In Colombia, 35 confirmed cases of neotropical polycystic echinococcosis were reported during 1978–2018. In most cases, *Echinococcus vogeli* was identified by means of morphologic identification. We describe a case of *E. vogeli* echinococcosis in a woman, diagnosed through PCR, mitochondrial DNA sequencing, and molecular characterization.

Human echinococcosis (also known as hydatidosis) is a zoonotic neglected disease caused by infection of cestode larval form of *Echinococcus* spp. tapeworms (*1*,*2*). At least 4 *Echinococcus* species are recognized as causes of human disease and have relevance in public health: *E. granulosus* causes cystic echinococcosis and is a cosmopolitan species; *E. multilocularis* produces alveolar echinococcosis and predominates in the northern hemisphere; and *E. vogeli* causes neotropical polycystic and *E. oligarthus* unicystic echinococcosis, both confined to tropical zones in Central and South America (*1*,*2*). Neotropical polycystic echinococcosis (NPE) affects mostly persons living in rural and sylvatic regions where the cycle of the *E. vogeli* tapeworm involves the paca (*Cuniculus paca*) as the intermediate host and the bush dog (*Speothus venaticus*) as the natural final host (*2*,*3*). Nevertheless, a proposed domiciliary transmission cycle posits that human infection occurs incidentally through fecal contamination by domestic hunting dogs (alternative final host) after they fed on paca viscera (*2*,*4*).

In Colombia, 35 confirmed cases of NPE were reported during 1978–2018 (*2*,*5*–*7*). In most of them, *Echinococcus* spp. infection was identified by means of morphologic identification (*2*,*5*,*6*); in 1 case, the 2018 report, molecular detection identified the *E. vogeli* cytochrome c oxidase subunit 1 (*cox1*) mitochondrial gene without molecular characterization (*7*). Here, we present a case of *E. vogeli* echinococcosis in a woman in Colombia diagnosed through PCR, sequencing mitochondrial DNA, and molecular characterization. We obtained written consent from the patient to report on her case. 

An otherwise healthy 50-year-old woman sought care at the emergency department of the Hospital Militar Central (Bogotá, Colombia) on September 23, 2024, after 8 days of epigastric and right hypochondrium pain; she did not have jaundice or other symptoms. As a child, she had lived in a rural region of Cesar Department (Colombian Caribbean region); she saw pacas often and always had kept domestic hunting dogs. Physical examination revealed a mild tenderness in right hypochondrium without peritoneal irritation signs. Serum liver function tests were without abnormality. Abdominal magnetic resonance imaging showed hypodense, round polycystic vesicles, replacing right liver parenchyma with predominant peripheral calcifications and fat content (Figure 1, panel A). Differential diagnoses were mucinous cystic neoplasm, hepatic liposarcoma, and hydatid cysts. We performed a total right segmental liver resection and cholecystectomy. The histopathological results of surgical liver specimen showed multiple cysts with *Echinococcus* protoscoleces (Figure 1, panel B). The patient received albendazole (200 mg 2×/d) for 1 month and was discharged. 

**Figure 1:**
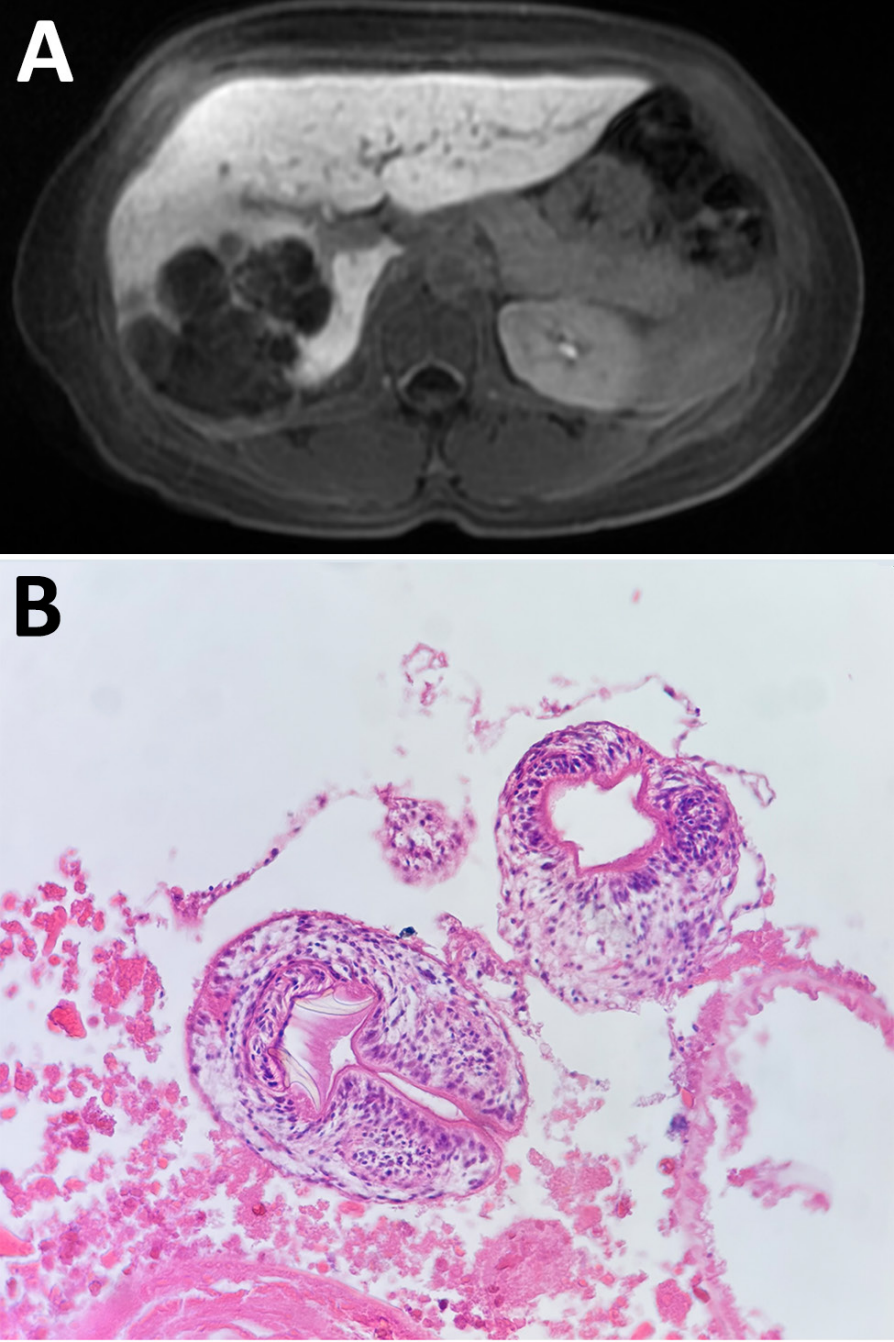
Neotropical polycystic echinococcosis in a woman, Colombia, 2024. A) Abdominal MRI scan showed hypodense, round polycystic vesicles, replacing right liver parenchyma with predominant peripheral calcifications and fat content. B) Protoscoleces of *Echinococcus* tapeworms with rostellar hooks. Hematoxylin & eosin staining; original magnification ×4.

We performed PCR on the liver histopathological sample, targeting the cytochrome b (Cob), 12S rRNA, and *cox1* genes, confirming the presence of *Echinococcus* sp. We sequenced the amplicons using Oxford Nanopore MinION (Oxford Nanopore Technologies, https://nanoporetech.com) and used those sequences for phylogenetic reconstruction. Phylogenetic analysis showed that the sequences we obtained of the 3 genes clustered with *E. vogeli* sequences downloaded from GenBank (Figure 2). Sequences from this study were deposited in GenBank (accession nos. PV243336, PV243987, and SUB15312175).

**Figure 2:**
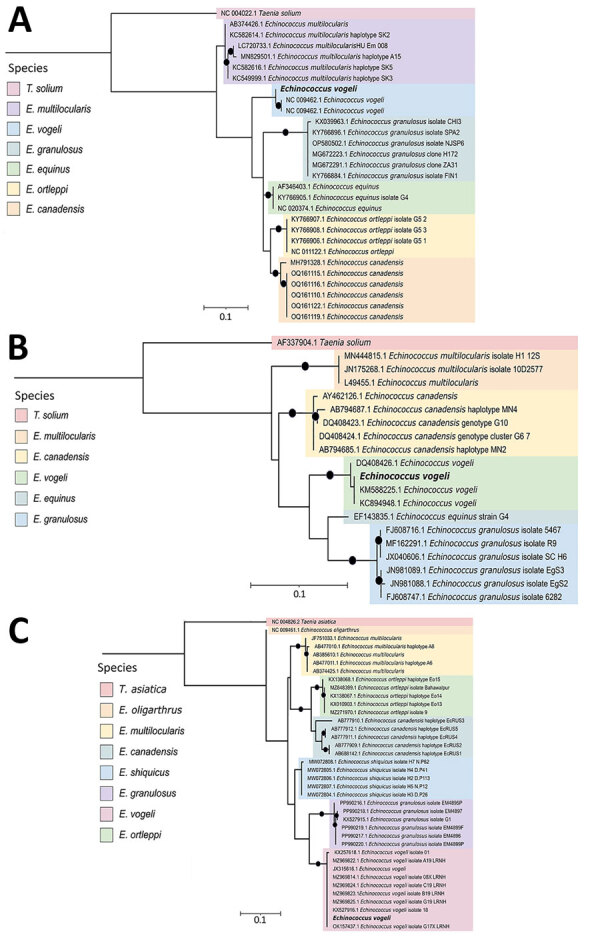
Phylogenetic reconstruction of the cytochrome b (A), 12S rRNA (B) and cytochrome c oxidase subunit 1 (C) genes of the *Taeniidae* family from consensus sequences obtained in study of *Echinococcus vogeli* infection in a human, Colombia, 2024. Bold text indicates strains from this study. Black dots indicate node support for bootstrap values >80. We used a maximum-likelihood approach to construct phylogenetic trees using IQ-TREE multicore version 1.6.12 (https://iqtree.github.io/release/v1.6.12). The best-fitting nucleotide substitution model for each gene was automatically selected by the software, and default parameters were applied. GenBank accession numbers are provided for reference sequences. Scale bar indicates substitutions per site.

The clinical characteristics of NPE in patients depend on the location of the metacestode as well as the extent of invasion of tissues (*2*). The liver is the most frequently affected organ (*2*). Metacestodes could be found in the liver alone or with vesicles situated in the abdomen, in the liver and the lungs or pleural cavities, or only as calcified vesicles in the liver (*2*). Other organs involved included the diaphragm, spleen, pancreas, omentum, mesenteries, rectovesical pouch, ovaries, uterus, abdominal wall, psoas muscle, and vertebra (*2*). Before surgery, patients often receive misdiagnosis with a variety of disorders, including hepatic tumor, abscess, cirrhosis or cholecystitis, gall bladder cancer, mesenteric tumor, and costal chondrosarcoma (*2*).

Geographic origin of the patients is a crucial diagnostic clue for *E. vogeli* echinococcosis (*2*). They are typically born in or have lived for prolonged periods in rural tropical areas of Central or South America, particularly in regions with abundant wildlife (*2*). Familiarity with pacas and whether domestic dogs were fed viscera of pacas are characteristics that contribute to a correct diagnosis (*2*). Ultrasound fine-needle aspiration and histopathologic examination of surgical specimens can permit taxonomic identification through larval morphologic clues (protoscoleces, hook shape and size, proportions of small and long blades) and are considered the standard, but definitive diagnosis is difficult when the hooks are absent (*1*–*3*). Since 2017, in Brazil, using molecular characterization of *E. vogeli* through *cox1* mitochondrial gene in samples from humans, domestic dogs, and pacas has suggested the presence of shared haplotypes among different populations of this cestode, reflecting the retention of ancestral polymorphisms (*8*–*10*).

In summary, our report highlights the value of molecular characterization of *E. vogeli* from histopathologic samples. Consistent with previous reports (*2*), we recommend that NPE be considered not as a medical curiosity but as a possible diagnosis of polycystic masses in humans from tropical zones in Central or South America.

## References

[1] Wen H, Vuitton L, Tuxun T, Li J, Vuitton DA, Zhang W, et al. Echinococcosis: Advances in the 21st Century. Clin Microbiol Rev. 2019;32:e00075–18. 10.1128/CMR.00075-18 30760475 PMC6431127

[2] D’Alessandro A, Rausch RL. New aspects of neotropical polycystic (*Echinococcus vogeli*) and unicystic (*Echinococcus oligarthrus*) echinococcosis. Clin Microbiol Rev. 2008;21:380–401. 10.1128/CMR.00050-07 18400802 PMC2292577

[3] Tappe D, Stich A, Frosch M. Emergence of polycystic neotropical echinococcosis. Emerg Infect Dis. 2008;14:292–7. 10.3201/eid1402.070742 18258123 PMC2600197

[4] D’Alessandro A, Rausch RL, Morales GA, Collet S, Angel D. *Echinococcus* infections in Colombian animals. Am J Trop Med Hyg. 1981;30:1263–76. 10.4269/ajtmh.1981.30.1263 7325284

[5] D’Alessandro A, Rausch RL, Cuello C, Aristizabal N. *Echinococcus vogeli* in man, with a review of polycystic hydatid disease in Colombia and neighboring countries. Am J Trop Med Hyg. 1979;28:303–17. 10.4269/ajtmh.1979.28.303 572148

[6] Botero D, Restrepo MI, Restrepo A. Report of four cases of neotropical polycystic equinococcosis caused by *Echinococcus vogeli* in Colombia. Curr Trop Med Rep. 2016;3:173–5. 10.1007/s40475-016-0090-2

[7] Mondragon-Cardona A, Duran-Gutierrez LF, Yucuma-Gutierrez S, Bolaños F, Vizcaychipi K, Alzate-Carvajal V, et al. Neotropical echinococcosis caused by *Echinococcus vogeli* in rural Huila, Colombia: report of two last cases in the last 35 years. Int J Infect Dis. 2018;73:306–7. 10.1016/j.ijid.2018.04.4112

[8] das Neves LB. Teixeira PEF, Silva S, de Oliveira FB, Garcia DD, de Almeida FB, et al. First molecular identification of *Echinococcus vogeli* and *Echinococcus granulosus* (sensu stricto) G1 revealed in feces of domestic dogs (*Canis familiaris*) from Acre, Brazil. Parasit Vectors. 2017;10:28. 10.1186/s13071-016-1952-0 PMC5237554 28088247

[9] Bittencourt-Oliveira F, Teixeira P, Alencar A, Menezes R, Corrêa C, Neves L, et al. First parasitological, histopathological and molecular characterization of *Echinococcus vogeli* Rausch and Bernstein, 1972 from *Cuniculus paca* Linnaeus, 1766 in the Cerrado biome (Mato Grosso do Sul, Brazil). Vet Parasitol. 2018;250:35–9. 10.1016/j.vetpar.2017.12.003 29329621

[10] Daipert-Garcia D, Pavan MG, Neves LBD, Almeida FB, Siqueira NG, Santos GBD, et al. Genetic diversity of *Echinococcus vogeli* in the western Brazilian Amazon. Mem Inst Oswaldo Cruz. 2019;114:e190149. 10.1590/0074-02760190149 31576902 PMC6764793

